# Risk factors and predictive model for postoperative cerebrospinal fluid leakage following endoscopic endonasal pituitary adenoma surgery: a retrospective study focusing on pneumocephalus and sellar floor bony window

**DOI:** 10.3389/fendo.2025.1695573

**Published:** 2025-10-31

**Authors:** Jiawei Zhang, Yurui He, Yijie Ning, Rui Bai, Hongqin Wang

**Affiliations:** ^1^ Department of Neurosurgery, The First Hospital of Shanxi Medical University, Taiyuan, China; ^2^ Department of Vascular Surgery, The Second Hospital of Shanxi Medical University, Taiyuan, China; ^3^ Vascular Institute of Shanxi Medical University, Taiyuan, China

**Keywords:** pneumocephalus, sellar floor bony window, cerebrospinal fluid leakage (CSF leakage), pituitary adenoma, predictive model

## Abstract

**Objective:**

To identify risk factors for cerebrospinal fluid (CSF) leakage after endoscopic endonasal pituitary adenoma surgery and to develop a clinical prediction model aimed at facilitating early detection and reducing the risk of related complications.

**Methods:**

Clinical data were retrospectively collected from patients who underwent endoscopic endonasal pituitary adenoma resection between January 2021 and September 2024. Postoperative CSF leakage was diagnosed based on clinical manifestations, glucose testing, and expert consensus. Univariable and multivariable logistic regression analyses were performed to determine independent risk factors, which were subsequently incorporated into a nomogram. The predictive performance of the model was evaluated using receiver operating characteristic (ROC) curves, calibration curves, and decision curve analysis (DCA).

**Results:**

Among 254 patients, 19 (7.48%) developed postoperative CSF leakage. Compared with those without leakage, affected patients exhibited larger tumor diameters, wider sellar floor bony windows, higher intraoperative CSF leak grades, greater suprasellar extension, and higher postoperative pneumocephalus grades. Multivariate logistic regression identified postoperative pneumocephalus grade (OR = 12.90, 95% CI: 3.24-59.80, *p* < 0.001), sellar floor bony window size (OR = 1.20, 95% CI: 1.07-1.37, *p* = 0.004), suprasellar extension grade (OR = 5.87, 95% CI: 1.15-46.10, *p* = 0.049), and intraoperative CSF leak grade (OR = 4.71, 95% CI: 1.14-20.30, *p* = 0.032) as independent predictors of postoperative CSF leakage. The nomogram incorporating these factors achieved excellent predictive accuracy, with an AUC of 0.948 (95% CI: 0.906–0.991).

**Conclusion:**

This study confirms that a higher grade of pneumocephalus and a larger sellar floor bony window are significant risk factors for postoperative CSF leak following endoscopic endonasal transsphenoidal surgery for pituitary tumors. Building on these findings, we developed a clinical prediction model for postoperative CSF leak by integrating relevant preoperative, intraoperative, and postoperative factors. This model facilitates the early prevention, identification, and management of CSF leaks, which is crucial for reducing the risk of associated complications and improving patient outcomes.

## 1 Introduction

Pituitary adenomas are benign tumors located in the sellar region and account for approximately 10-15% of all intracranial space-occupying lesions (1). With advances in imaging and surgical techniques, the endoscopic endonasal approach has become the preferred treatment for most pituitary adenomas (2). However, due to the thin skull base bone and complex anatomical structures, complications such as cerebrospinal fluid (CSF) leakage, diabetes insipidus, and hypopituitarism remain relatively common (3, 4). Among these, postoperative CSF leakage is particularly concerning, as it may lead to a range of secondary complications, including intracranial hypotension, meningitis, and tension pneumocephalus, which in severe cases can even be life-threatening (5).

Intracranial pneumocephalus is a common complication following endoscopic endonasal skull base surgery (6). Clinical manifestations can include headache, visual disturbances, and in severe cases, tension pneumocephalus, which may lead to rapid neurological deterioration and even threaten life (7). Recent studies have indicated that both the extent and distribution of intracranial air are closely associated with postoperative CSF leakage (6, 8), yet few reports have incorporated these parameters into clinical prediction models. The sellar floor bony window refers to the area of bone removal during endoscopic endonasal surgery, reflecting the degree of bony destruction and the technical difficulty of skull base reconstruction (9). Although its precise role in postoperative CSF leakage has not been fully elucidated, several studies suggest that the integrity of the sellar floor is crucial for supporting reconstructive materials during skull base repair (9, 10).

Building upon previously reported risk factors, we further evaluated variables such as pneumocephalus grade and the size of the sellar floor bony window to more accurately quantify the potential risks for CSF leak following endoscopic endonasal pituitary adenectomy. We subsequently developed and validated a clinical prediction model for postoperative CSF leak. This model aims to facilitate the early identification, management, and intervention in high-risk patients, thereby reducing the incidence of postoperative complications and improving patient outcomes. Furthermore, we hypothesize that patients with a larger intraoperative sellar floor bony window and a higher postoperative pneumocephalus grade are at a significantly increased risk of developing a CSF leak.

## 2 Methods

### 2.1 Study population

This retrospective study was approved by the Ethics Committee of the First Hospital of Shanxi Medical University. Clinical data from 254 patients who underwent endoscopic endonasal surgery for pituitary adenomas in the Department of Neurosurgery between January 2021 and September 2024 were analyzed. Inclusion criteria were: (1) age ≥ 18 years; (2) preoperative cranial MRI diagnosis of pituitary adenoma and planned endoscopic endonasal resection, with postoperative pathological confirmation of pituitary neuroendocrine tumor. Exclusion criteria were: (1) history of craniotomy for pituitary tumor resection; (2) presence of severe cardiovascular, cerebrovascular, or malignant diseases; and (3) incomplete clinical or imaging data (with pre- and postoperative sellar MRI and a 24-hour postoperative CT scan). Data extraction was performed by one medical staff member and verified for statistical analysis by another. All patients or their legal guardians provided informed consent for surgery, and the study was conducted in accordance with the Declaration of Helsinki (**Supplementary Figure 1** shows a typical case of postoperative cerebrospinal fluid leakage).

### 2.2 Study variables

The primary variables included patient demographics, comorbidities, tumor characteristics (tumor size, Knosp grade, suprasellar extension grade, pathological type), intraoperative parameters (extended approach, sellar floor bony window size, intraoperative CSF leak grade, tumor consistency, extent of tumor resection), and postoperative pneumocephalus grade. The maximum diameter of the sellar floor drilled, which was measured jointly by the primary surgeon and the first assistant with all results reaching consensus and being recorded in the operative notes, was documented as the bony window size. The extent of tumor resection was determined based on intraoperative assessment and postoperative sellar MRI. Tumor consistency and use of a nasoseptal flap were obtained from operative records. Suprasellar extension was classified according to Hardy grading. Tumor consistency was assessed using the Rutkowski grading system (11). Intraoperative CSF leakage was graded using the Kelly system (12), and pneumocephalus grade was assessed on routine CT scans performed within 24 hours postoperatively, according to the Banu grading system. (6) (**Supplementary Tables 2**-**4**).

### 2.3 Skull base dural reconstruction and diagnosis of postoperative CSF leakage

Skull base dural reconstruction was performed according to the intraoperative Kelly grading system. For patients classified as Kelly grade 0 or 1, a unilateral reconstruction strategy was typically employed, such as coverage with a single layer of artificial dura mater. For those with Kelly grade 2 or 3, a multilayer reconstruction technique was adopted, involving the use of artificial dura, fat grafts, fascia lata, or a vascularized nasoseptal flap, with the placement sequence adjusted as appropriate based on intraoperative conditions. Notably, prophylactic lumbar drainage (LD) was not implemented for patients with Kelly grade 2 or 3.

Postoperative CSF leakage was diagnosed when any of the following criteria were met:(1) persistent clear fluid drainage from the nasal cavity with a positive glucose test result; (2) absence of persistent clear nasal discharge but a positive glucose test result confirmed by repeated testing, with the final diagnosis established by two experienced skull base surgeons (HW and RB, each with more than five years of neuroendoscopic surgical experience).Patients without nasal discharge and with negative glucose test results were classified as having no postoperative CSF leakage.

### 2.4 Feature analysis

Preoperative, intraoperative, and postoperative factors potentially associated with postoperative CSF leakage were analyzed. Continuous variables with a normal distribution were expressed as mean ± standard deviation ( $\bar{\text{x}}$ ± s) and compared between groups using the independent-samples *t*-test. Non-normally distributed continuous variables were presented as median (*Q1*, *Q3*) and compared using the Mann–Whitney *U* test. Categorical variables were summarized as counts and percentages, and differences between groups were assessed using the chi-square test. Factors showing statistical significance in univariate logistic regression were further evaluated using receiver operating characteristic (ROC) curves and included in a multivariable binary logistic regression model to identify independent predictors of postoperative CSF leakage. A *p*-value < 0.05 was considered statistically significant. All statistical analyses were performed using R software (version 4.4.1).

### 2.5 Predictive model development:

Independent risk factors identified from multivariable logistic regression (p < 0.05) were incorporated into a binary logistic regression model to construct a predictive nomogram using R software. Model performance was assessed by calibration curves and decision curve analysis (DCA), and discriminative ability was evaluated using ROC curves. The ROC and calibration curves quantified the predictive accuracy, while the DCA curve assessed the clinical utility of the model.

## 3 Results

### 3.1 Baseline characteristics:

A total of 254 patients who underwent endoscopic endonasal pituitary adenoma resection were included in this study (**Table 1**). Patients were classified into two groups according to the presence or absence of postoperative CSF leakage. Among them, 19 patients (7.48%) developed postoperative CSF leakage during hospitalization. All affected patients were managed with conservative measures, including strict supine bed rest, therapeutic lumbar drainage, and prophylactic antibiotic therapy. Following these interventions, all patients demonstrated clinical improvement and were discharged in stable condition. The median age (interquartile range) was 58 (37, 63) years in the CSF leakage group and 55 (47, 63) years in the non-leakage group (*p* = 0.798). The median body mass index (BMI) was 23.31 (22.04, 25.39) in the leakage group and 24.97 (22.49, 27.34) in the non-leakage group (*p* = 0.063). Tumor maximum diameter was significantly larger in the leakage group (2.8 cm [2.5, 4.0]) compared with the non-leakage group (2.5 cm [2.0, 3.1], *p* = 0.038). The median sellar floor bony window size was 30 mm (25, 30) in the leakage group versus 20 mm (15, 25) in the non-leakage group (*p* < 0.001).

**Table 1:** Baseline characteristics of pituitary adenoma patients with and without postoperative cerebrospinal fluid leakage.

Characteristics	Total (*n* = 254)	No CSF leak (*n* = 235)	CSF leak (*n* = 19)	*P-value*
Age (year)	55.0 (47.0,63.0)	55.0 (47.0,63.0)	58.0 (37.0,63.0)	0.798
Male (%)	135.0 (53.1)	126.0 (53.6)	9.0 (47.4)	0.600
BMI (kg/m^2^)	24.8 (22.5,27.2)	25.0 (22.5,27.3)	23.3 (22.0,25.4)	0.063
Hypertension Yes (%)	79.0 (31.1)	72.0 (30.6)	7.0 (36.8)	0.574
Diabetes Yes (%)	29.0 (11.4)	27.0 (11.5)	2.0 (10.5)	0.899
Pathology type (%)				0.855
Non-functional adenoma	231.0 (90.9)	213.0 (90.6)	18.0 (94.7)	
Functional adenoma	23.0 (9.1)	22.0 (9.4)	1.0 (5.3)	
Recurrence Yes (%)	35.0 (13.8)	31.0 (13.2)	4.0 (21.1)	0.542
Maximum diameter (cm)	2.6 (2.0,3.1)	2.5(2.0,3.1)	2.8 (2.5,4.0)	0.038
Vertical diameter (cm)	2.4 (1.8,2.9)	2.3 (1.7,2.9)	2.6 (2.5,3.0)	0.039
Transverse diameter (cm)	2.2 (1.8,2.7)	2.2 (1.8,2.7)	2.4 (2.1,3.1)	0.044
Anteroposterior diameter (cm)	1.9 (1.5,2.3)	1.8 (1.5,2.3)	2.1 (1.8,2.6)	0.015
Knosp grading (%)				0.824
0	25.0 (9.8)	23.0 (9.8)	2.0 (10.5)	
1	34.0 (13.4)	31.0 (13.2)	3.0 (15.8)	
2	29.0 (11.4)	26.0 (11.1)	3.0 (15.8)	
3	92.0 (36.2)	87.0 (37.0)	5.0 (26.3)	
4	74.0 (29.1)	68.0 (28.9)	6.0 (31.6)	
Sellar floor bony window (cm)	2.0 (1.5,2.7)	2.0 (1.5,2.5)	3.0 (2.5,3.0)	<0.001
Extended approach Yes (%)	70.0 (27.6)	64.0 (27.2)	6.0 (31.6)	0.683
Kelly grading (%)				<0.001
0	163.0 (64.2)	163.0 (69.4)	0.0 (0.0)	
1	62.0 (24.4)	54.0 (23.0)	8.0 (42.1)	
2	16.0 (6.3)	12.0 (5.1)	4.0 (21.1)	
3	13.0 (5.1)	6.0 (2.6)	7.0 (36.8)	
Suprasellar extension grade (%)				<0.001
0	57.0 (22.4)	57.0 (24.3)	0.0 (0.0)	
A	113.0 (44.5)	111.0 (47.2)	2.0 (10.5)	
B	70.0 (27.6)	62.0 (26.4)	8.0 (42.1)	
C	14.0 (5.5)	5.0 (2.1)	9.0 (47.4)	
Pneumocephalus grade (%)				<0.001
0	99.0 (39.0)	99.0 (42.1)	0.0 (0.0)	
1	72.0 (28.3)	72.0 (30.6)	0.0 (0.0)	
2	52.0 (20.5)	46.0 (19.6)	6.0 (31.6)	
3	15.0 (5.9)	11.0 (4.7)	4.0 (21.1)	
4	16.0 (6.3)	7.0 (3.0)	9.0 (47.4)	
Tumor consistency (%)				0.374
1	3.0 (1.2)	2.0 (0.9)	1.0 (5.3)	
2	99.0 (39.0)	90.0 (38.3)	9.0 (47.4)	
3	117.0 (46.1)	110.0 (46.8)	7.0 (36.8)	
4	31.0 (12.2)	29.0 (12.3)	2.0 (10.5)	
5	4.0 (1.6)	4.0 (1.7)	0.0 (0.0)	
Gross total resection (%)	228.0 (89.8)	211.0 (89.8)	17.0 (89.5)	1.000
Nasoseptal flap Yes (%)	88.0 (34.6)	79.0 (33.6)	9.0 (47.4)	0.226

Regarding intraoperative CSF leak (Kelly grading), 0-level was observed in 163 patients (all non-leakage, 69.4%), 1-level in 62 patients (leakage group 8, 42.1%), 2-level in 16 patients (leakage group 4, 21.1%), and 3-level in 13 patients (leakage group 7, 36.8%). Suprasellar extension grades were distributed as follows: 0-level, 57 patients (all non-leakage); A-level, 113 patients (leakage group 2, 10.5%); B-level, 70 patients (leakage group 8, 42.1%); C-level, 14 patients (leakage group 9, 47.4%). Postoperative pneumocephalus grades were: 0-level, 99 patients (all non-leakage); 1-level, 72 patients (all non-leakage); 2-level, 52 patients (leakage group 6, 31.6%); 3-level, 15 patients (leakage group 4, 21.1%); 4-level, 16 patients (leakage group 9, 47.4%). Complete tumor resection was achieved in 211 patients (89.8%) in the non-leakage group and 17 patients (89.5%) in the leakage group.

### 3.2 Univariate and multivariate logistic regression analysis

Univariate logistic regression (**Table 2**) indicated that age, sex, BMI, hypertension, diabetes, pathological type, repeat surgery, extended approach, extent of tumor resection, nasoseptal flap use, Knosp grade, and tumor consistency were not significantly associated with postoperative CSF leakage. Tumor maximum diameter, vertical diameter, anterior-posterior diameter, transverse diameter, Kelly grade, suprasellar extension, pneumocephalus grade, and sellar floor bony window size showed significant differences between groups. Multivariate logistic regression identified the following independent predictors: Kelly grade (OR = 4.71, 95% CI: 1.14-20.30, *p* = 0.032), suprasellar extension grade (OR = 5.87, 95% CI: 1.15-46.10, *p* = 0.049), pneumocephalus grade (OR = 12.90, 95% CI: 3.24-59.80, *p* < 0.001), and sellar floor bony window size (OR = 1.20, 95% CI: 1.07-1.37, *p* = 0.004).

**Table 2:** Univariate and multivariate logistic regression analysis.

Characteristics	Univariate		Multivariate	
	OR (95%CI)	*p* Value	OR (95%CI)	*p* Value
Age	0.991 (0.956, 1.030)	0.635		
Sex, Male	0.779 (0.299, 2.000)	0.600		
BMI	0.888 (0.771, 1.010)	0.086		
Hypertension, Yes	1.320 (0.474, 3.420)	0.575		
Diabetes, Yes	0.906 (0.139, 3.400)	0.899		
Pathology, Functional	0.538 (0.029, 2.810)	0.555		
Reoperation, Yes	1.750 (0.477, 5.210)	0.344		
Maximum diameter	1.840 (1.180, 2.870)	0.007	0.322 (0.024, 3.790)	0.384
Vertical diameter	1.050 (1.010, 1.100)	0.023	1.050 (0.866, 1.300)	0.616
Transverse diameter	1.100 (1.040, 1.160)	0.001	1.140 (0.949, 1.390)	0.169
Anteroposterior diameter	1.110 (1.040,1.180)	0.002	1.010 (0.859, 1.200)	0.872
Extended approach, Yes	1.230 (0.418, 3.260)	0.684		
Kelly grade, ≥2	17.60(6.34, 51.50)	<0.001	4.710 (1.140, 20.300)	0.032
Suprasellar extension grades, ≥B	21.30 (5.90, 137.0)	<0.001	5.870 (1.150, 46.100)	0.049
Pneumocephalus grade, ≥3	26.10 (9.22, 82.40)	<0.001	12.90 (3.240, 59.80)	<0.001
Complete removal, Yes	0.967 (0.255, 6.330)	0.965		
Sellar floor bony window	1.170 (1.080, 1.290)	<0.001	1.200 (1.070, 1.370)	0.004
Nasoseptal flap, Yes	1.780 (0.680, 4.580)	0.231		
Knosp, ≥3	0.710 (0.276, 1.900)	0.479		
Consistency, ≥4	0.720 (0.111, 2.670)	0.670		

### 3.3 ROC curve analysis:

**Figure 1:**
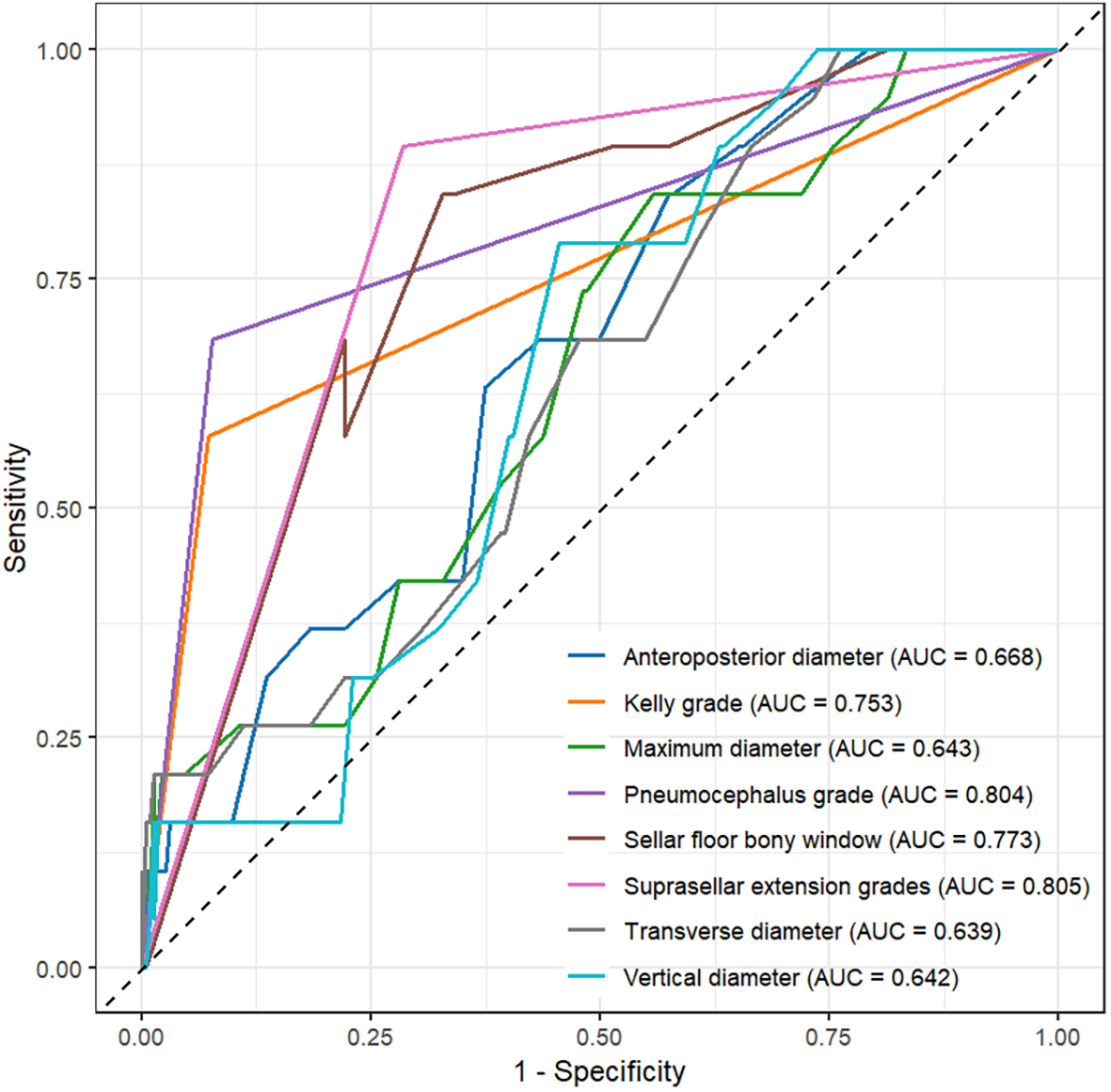
ROC curve analysis of maximum diameter, vertical diameter, transverse diameter, anteroposterior diameter, Kelly grade, suprasellar extension grade, pneumocephalus grade, and sellar floor opening size.

Factors showing significant differences in univariate logistic regression were further evaluated using ROC curves (**Figure 1**). The area under the curve (AUC) values were as follows: tumor maximum diameter 0.643, vertical diameter 0.642, transverse diameter 0.639, anterior-posterior diameter 0.668, Kelly grade 0.753, suprasellar extension 0.805, pneumocephalus grade 0.804, and sellar floor bony window size 0.773 (**Supplementary Table 1**).

### 3.4 Predictive model construction and evaluation

**Figure 2:**
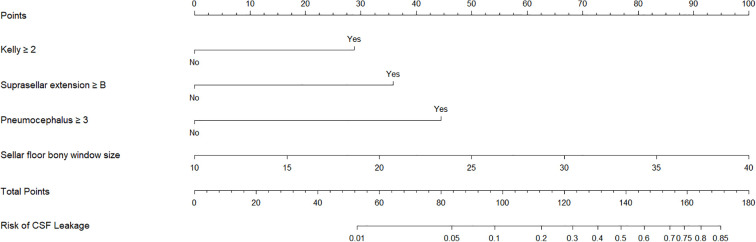
Nomogram construction. Nomogram constructed based on kelly grade, suprasellar extension grade, pneumocephalus grade, and sellar floor bony window.

**Figure 3:**
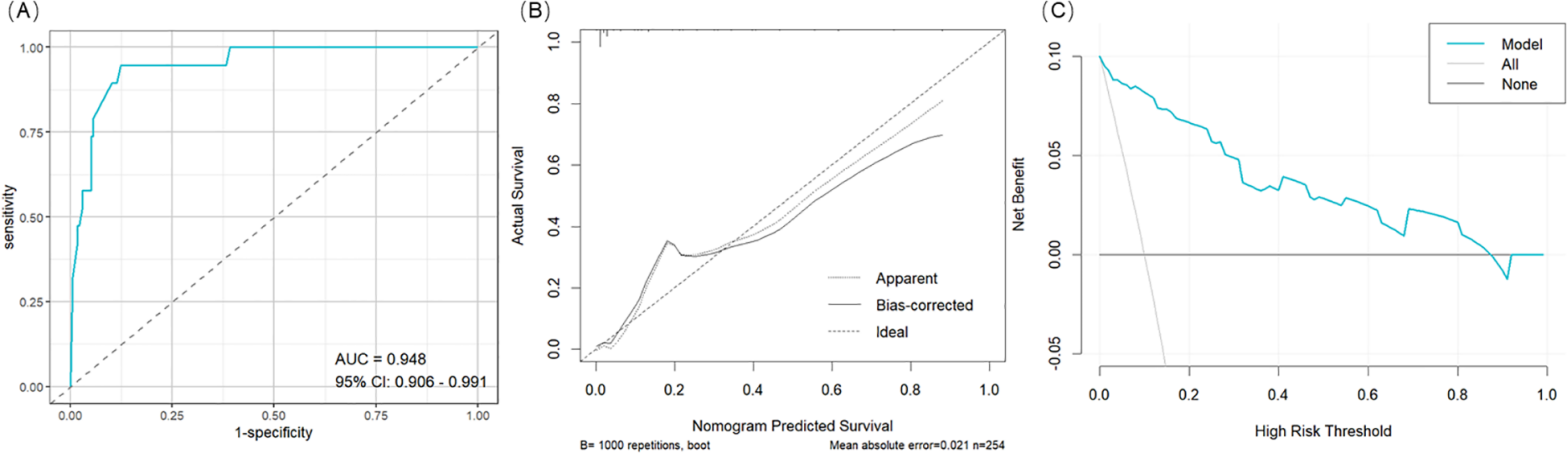
Nomogram Evaluation. **(A)** The ROC curve for the nomogram. **(B)** The calibration for the nomogram. **(C)** The decision curve analysis (DCA) for the nomogram.

A clinical prediction model was developed and presented as a nomogram based on the results of multivariate logistic regression analysis (**Figure 2**). The scoring system incorporated four parameters. The size of the sellar floor bony window was determined intraoperatively by measuring the maximal diameter of the drilled area. The degree of suprasellar extension was evaluated on preoperative imaging. The intraoperative Kelly grade was assessed during the surgical procedure, and the grade of pneumocephalus was determined using postoperative CT scans. Each parameter was assigned a specific score according to its regression coefficient, and the total score was calculated by summing the individual scores. The total point axis of the nomogram corresponded to the bottom scale, which represented the predicted risk of postoperative CSF leakage. The ROC curve demonstrated excellent discriminative ability with an AUC of 0.948 (95% CI: 0.906-0.991) (**Figure 3A**). Calibration curves showed that the bias-corrected line closely approximated the ideal line, indicating good predictive accuracy (**Figure 3B**). Decision curve analysis (DCA) indicated that, for threshold probabilities of 0-80%, using the nomogram provided greater net benefit compared with treating all or no patients (**Figure 3C**).

## 4 Discussion

Postoperative cerebrospinal fluid (CSF) leakage is a significant adverse event following transsphenoidal pituitary adenoma surgery (13), with reported incidence ranging from 2% to 15% (14–16). It can lead to serious complications such as postoperative intracranial infection and tension pneumocephalus (5, 17), thereby prolonging hospital stay, adversely affecting patient prognosis, and increasing healthcare costs (18). In the present study, 7.48% of patients experienced postoperative CSF leakage, consistent with findings from a recent systematic review (19).

Our findings identified intraoperative CSF leak grade, suprasellar tumor extension, size of the sellar floor bony window, and postoperative pneumocephalus grade as significant factors influencing postoperative CSF leakage. The intraoperative CSF leak grade has long been recognized as a critical predictor, with studies reporting that patients with intraoperative CSF leaks are 6.33 times more likely to develop postoperative CSF leakage than those without (20, 21). Suprasellar tumor extension has also been reported as a key risk factor for postoperative CSF leakage (22). Anatomically, this association may be explained by the proximity of the pituitary tumor to the diaphragma sellae and arachnoid membrane: the superior portion of the sella is formed by a dural diaphragm, above which lie the arachnoid and pia mater, with CSF residing in the intervening space (23). Greater suprasellar extension increases the contact area between the tumor and arachnoid, raising the risk of arachnoid injury during surgical dissection, consistent with the “sellar barrier” principle described by Villalonga (24). The influence of tumor size on postoperative CSF leakage remains controversial. Some studies suggest that giant adenomas are more prone to CSF leakage than microadenomas or macroadenomas (18), whereas others report no correlation between tumor size and CSF leakage (25). In our study, although maximum tumor diameter and three linear dimensions differed significantly between groups, they were not statistically significant in multivariate analysis. This may reflect that suprasellar extension more directly represents the extent and intensity of tumor-arachnoid contact than tumor diameter alone (24).Intraoperative CSF leakage has been consistently associated with postoperative CSF leakage (26), and multiple studies emphasize controlling intraoperative CSF leak grade as a key preventive strategy (24, 27, 28).The intraoperative CSF leak grade reflects both the extent of arachnoid disruption and the volume of CSF flow (12). Higher-grade leaks often necessitate complex reconstruction using multiple graft materials combined with nasoseptal flaps (29), which not only complicates skull base repair but also increases the risk of postoperative CSF leakage.

In recent years, no studies have explicitly reported intraoperative sellar floor window size as a risk factor for postoperative CSF leakage. However, Li et al. (22) reported that the degree of tumor invasion into the sellar floor significantly influences postoperative CSF leakage. Similarly, Conger et al.in a large retrospective study (9), emphasized that excessive exposure of the lesion during surgery should be avoided to prevent extensive bony defects, which can complicate skull base reconstruction by hindering graft support within the sphenoid sinus and thereby increasing the risk of postoperative CSF leakage. These findings highlight the importance of sellar floor window size for effective skull base reconstruction. In this study, we quantified the sellar floor window and included it in the analysis of factors affecting postoperative CSF leakage. Our results demonstrated that larger window sizes were associated with higher postoperative CSF leak risk and exhibited good predictive performance in ROC analysis. Although the precise mechanism by which a larger sellar floor window increases CSF leak risk remains unclear, it is likely related to the increased difficulty of skull base reconstruction. Specifically, a larger bony window reduces the available bony support, increasing the risk of graft displacement (10) and complicating the adhesion of reconstructive materials and mucosal flaps.

Intracranial pneumocephalus is observed in 30-40% of patients following endoscopic transnasal surgery, with a markedly higher prevalence in those with pituitary adenomas (57.14%) (17, 30). Both the grade and distribution of pneumocephalus are closely associated with the risk of postoperative CSF leakage (6, 8). To our knowledge, this study is the first to incorporate pneumocephalus grading into a clinical prediction model for postoperative CSF leakage, and the model maintained good predictive performance. Using the Banu classification, we evaluated pneumocephalus (6) and found that postoperative pneumocephalus grade demonstrated the strongest predictive effect for CSF leakage (AUC = 0.804), playing a critical role in the overall accuracy of the model. The association between pneumocephalus and postoperative CSF leakage may be explained by two mechanisms: the ball-valve mechanism and the inverted bottle mechanism (31–33). Gas can enter the sellar, parasellar, and convexity regions through gaps between the sellar floor and reconstruction materials, but it is difficult to evacuate. In addition, CSF leakage from a disrupted arachnoid generates negative pressure in the subarachnoid space, further facilitating gas entry. These mechanisms indicate that pneumocephalus grade reflects both the integrity of sellar floor reconstruction and the extent of suprasellar arachnoid disruption, which likely accounts for its strong predictive value for postoperative CSF leakage.

Based on univariate and multivariate logistic regression analyses, we constructed a nomogram integrating independent risk factors to predict the probability of postoperative CSF leakage. The scoring system incorporates four variables: preoperative assessment of suprasellar tumor extension (≥grade B), intraoperative measurement of sellar floor window size, intraoperative CSF leak grade (≥grade 2), and postoperative 24-hour pneumocephalus grade (≥grade 3). Each variable contributes a corresponding score, and the sum of these scores corresponds to the predicted probability of postoperative CSF leakage, ranging from 0.01 to 0.85. This nomogram enables probability-based risk prediction across preoperative, intraoperative, and postoperative dimensions. The predictive performance of the model was evaluated using ROC, calibration, and decision curve analyses (DCA), all indicating good clinical applicability. The nomogram provides a practical tool for early identification of patients at high risk of postoperative CSF leakage and can inform clinical decision-making.

This study confirms that a larger sellar floor bony window and a higher grade of pneumocephalus are significantly associated with an increased risk of postoperative CSF leakage, which is consistent with our initial hypothesis. Removal of the sellar bone is an essential step in transsphenoidal surgery (29). A larger bony window often indicates a thinner sellar floor structure and consequently increases the difficulty of skull base reconstruction (9, 10). Therefore, based on preoperative imaging findings regarding tumor size, location, and invasiveness, the bony window should be carefully controlled to minimize the extent of bone removal while ensuring adequate tumor exposure. Additionally, a multi-layer skull base reconstruction strategy is recommended. For instance, Esposito et al. proposed a tailored multi-layer repair protocol based on the intraoperative Kelly grading system (12), and the “3F” reconstruction strategy introduced by Solari et al. is also noteworthy (34).Furthermore, the pneumocephalus grade may reflect the integrity of skull base reconstruction and the extent of diaphragmatic sellae disruption, serving as an early indicator for postoperative CSF leakage. Recent studies have shown that prophylactic lumbar drainage (LD) is widely adopted by skull base surgeons to reduce the risk of CSF leakage (35–37). A randomized controlled trial further demonstrated the beneficial role of LD in decreasing the incidence of postoperative CSF leakage (38). However, another randomized study reported no significant difference in CSF leak rates between patients who received prophylactic LD and those who did not, while the LD group experienced a higher rate of complications. These findings suggest that the clinical efficacy of prophylactic LD remains uncertain and warrants further high-quality prospective investigation. In our center’s experience, for high-risk patients—such as those requiring extensive removal of the sellar floor bone or presenting with severe postoperative pneumocephalus—multilayer skull base reconstruction is the key strategy for preventing postoperative CSF leakage, whereas the added benefit of prophylactic LD still requires additional clinical validation. In contrast, when LD is used as a therapeutic intervention in patients who have already developed postoperative CSF leaks, it can effectively lower intracranial pressure, reduce tension at the repair site, and thereby promote sellar floor healing while minimizing associated complications. Therefore, the use of LD should be individualized and selectively applied, taking into account patient-specific risk factors and intraoperative findings.

However, this study has several limitations. First, as a retrospective single-center study with a limited sample size, the results may be subject to inherent bias, and individualized risk assessment is recommended for different populations. Second, the limited sample size precluded the creation of internal and external validation sets to assess the model’s predictive accuracy. Future studies will expand the sample size and incorporate multicenter data to enhance the reliability and generalizability of the model.

## 5 Conclusion

This study identified suprasellar tumor extension, sellar floor window size, intraoperative CSF leak grade, and postoperative pneumocephalus grade as independent risk factors for postoperative CSF leakage following endoscopic transnasal pituitary adenoma resection. Based on these factors, we developed a nomogram prediction model, which demonstrated good usability and reliability. The model effectively predicts postoperative CSF leakage risk and facilitates early identification of high-risk patients. It provides clinicians with a valuable tool to implement timely preventive and interventional strategies, potentially reducing the incidence of CSF leakage and related complications, and improving patient outcomes.

## Data Availability

The raw data supporting the conclusions of this article will be made available by the authors, without undue reservation.
