# Supplementary material for: Risk factors and predictive model for postoperative cerebrospinal fluid leakage following endoscopic endonasal pituitary adenoma surgery: a retrospective study focusing on pneumocephalus and sellar floor bony window
Source: Front Endocrinol (Lausanne). 2025 Oct 31;16:1695573. doi: 10.3389/fendo.2025.1695573 (PMC12615192; doi:10.3389/fendo.2025.1695573)
Supplement: Supplementary file 1 [file DataSheet1.docx]

Supplementary Material

**Supplementary Table 1.** ROC curve analysis of maximum diameter, vertical diameter, transverse diameter, anteroposterior diameter, Kelly grade, suprasellar extension grade, pneumocephalus grade, and sellar floor opening size.

| **Characteristics** | **Cut off** | **AUC** | **Sensitivity** | **Specificity** | **Youden**  **(max)** | **95% CI** | ***P*** |
| --- | --- | --- | --- | --- | --- | --- | --- |
| Maximum diameter | 2.45 | 0.643 | 0.842 | 0.443 | 0.285 | (0.520, 0.766) | 0.384 |
| Vertical diameter | 24.5 | 0.642 | 0.789 | 0.545 | 0.334 | (0.538, 0.746) | 0.616 |
| Transverse diameter | 17.5 | 0.639 | 1 | 0.238 | 0.238 | (0.519, 0.758) | 0.169 |
| Anteroposterior diameter | 17.65 | 0.668 | 0.842 | 0.426 | 0.268 | (0.555, 0.781) | 0.872 |
| Kelly grade,≥2 | 1.5 | 0.753 | 0.579 | 0.928 | 0.507 | (0.638, 0.869) | 0.032 |
| Suprasellar extension grades,≥B | 1.5 | 0.805 | 0.845 | 0.715 | 0.61 | (0.728, 0.881) | 0.049 |
| Pneumocephalus grade，≥3 | 1.5 | 0.804 | 0.684 | 0.923 | 0.608 | (0.695, 0.913) | <0.001 |
| Sellar floor opening size | 24.5 | 0.773 | 0.842 | 0.672 | 0.514 | (0.682, 0.863) | 0.004 |

**Supplementary Table 2.** Cerebrospinal fluid leak grading system (Kelly system)

| Grade of leak | Description of leak |
| --- | --- |
| Grade 0 | Absence of cerebrospinal fluid leak, confirmed by Valsalva maneuver |
| Grade 1 | Small “weeping” leak, confirmed by Valsalva maneuver, without obvious or with only small diaphragmatic defect |
| Grade 2 | Moderate cerebrospinal fluid leak, with obvious diaphragmatic defect |
| Grade 3 | Large cerebrospinal fluid leak, typically created as part of extended transsphenoidal approach through the supradiaphragmatic or clival dura for tumor access |

**Supplementary Table 3.** Rutkowski grading system

| Consistency Grade | Description |
| --- | --- |
| Grade 1 | Cystic or hemorrhagic tumor consistency |
| Grade 2 | Soft tumor consistency; freely suckable tumor; minimal curettage required |
| Grade 3 | Average tumor consistency; partially suckable tumor, requires some curettage or mechanical debulking; tumor readily descends from suprasellar space |
| Grade 4 | Firm tumor consistency; not suckable, curettage or mechanical debulking required; tumor does not readily descend from suprasellar space; extracapsular technique typically required |
| Grade 5 | Extremely firm or calcified tumor; not curettable, requires sharp or en bloc removal |

**Supplementary Table 4.** Grade of pneumocephalus (Banu system)

| Grade of pneumocephalus | Description |
| --- | --- |
| Grade 0 | none |
| Grade 1 | dots of air (< 1 mm of air) |
| Grade 2 | bubbles (< 1 cm of air) |
| Grade 3 | 1–3 cm of air |
| Grade 4 | > 3 cm of air |

**Supplementary Figure 1.** Preoperative and postoperative imaging and pathological findings of a patient who developed postoperative CSF leakage.


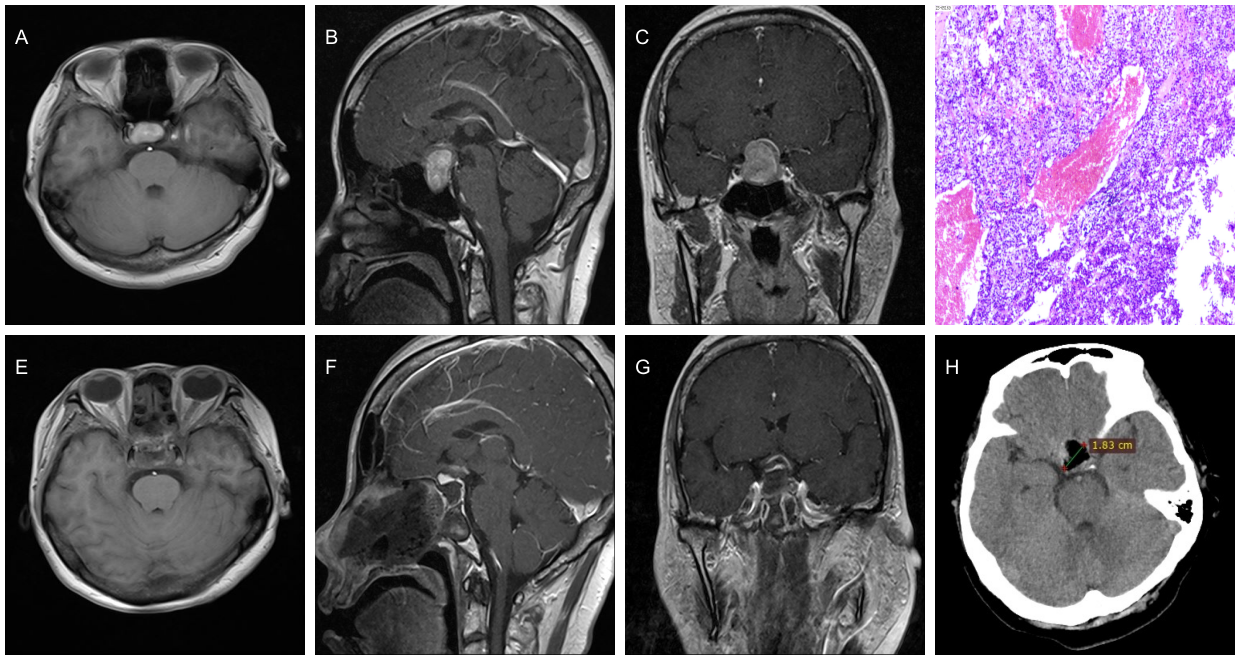


A–C: Preoperative contrast-enhanced MRI of the sellar region (suprasellar extension grade B);

D: Histopathological findings showing a PitNET, null cell type;

E–G: Postoperative contrast-enhanced MRI of the sellar region (complete tumor resection);

H: CT scan within 24 hours after surgery (pneumocephalus grade 3).
